# Metagenomic evidence for a polymicrobial signature of sepsis

**DOI:** 10.1099/mgen.0.000642

**Published:** 2021-09-03

**Authors:** Cedric Chih Shen Tan, Mislav Acman, Lucy van Dorp, Francois Balloux

**Affiliations:** ^1^​ UCL Genetics Institute, University College London, Gower Street, London, WC1E 6BT, UK; ^2^​ Genome Institute of Singapore, A*STAR, Singapore 138672, Singapore

**Keywords:** bacteraemia, machine learning, metagenomics, sepsis, contamination, kitome, blood metagenomics, SHAP

## Abstract

Our understanding of the host component of sepsis has made significant progress. However, detailed study of the microorganisms causing sepsis, either as single pathogens or microbial assemblages, has received far less attention. Metagenomic data offer opportunities to characterize the microbial communities found in septic and healthy individuals. In this study we apply gradient-boosted tree classifiers and a novel computational decontamination technique built upon SHapley Additive exPlanations (SHAP) to identify microbial hallmarks which discriminate blood metagenomic samples of septic patients from that of healthy individuals. Classifiers had high performance when using the read assignments to microbial genera [area under the receiver operating characteristic (AUROC=0.995)], including after removal of species ‘culture-confirmed’ as the cause of sepsis through clinical testing (AUROC=0.915). Models trained on single genera were inferior to those employing a polymicrobial model and we identified multiple co-occurring bacterial genera absent from healthy controls. While prevailing diagnostic paradigms seek to identify single pathogens, our results point to the involvement of a polymicrobial community in sepsis. We demonstrate the importance of the microbial component in characterising sepsis, which may offer new biological insights into the aetiology of sepsis, and ultimately support the development of clinical diagnostic or even prognostic tools.

## Data Summary

All relevant source code and parsed datasets used can be found on GitHub (https://github.com/cednotsed/Polymicrobial-Signature-of-Sepsis). The raw sequence data for each study can be found from NCBI SRA and the European Nucleotide Archive repository with the accessions listed in Table 1. The authors confirm all supporting data, code and protocols have been provided within the article or through supplementary data files.

**Table 1.** Summary of metagenomic datasets. Sample sizes indicated here are those after all quality control steps have been applied. Grumaz-16/19 is a combined dataset comprising Grumaz-16 and Grumaz-19.

Study	Dataset alias	Accession	Sepsis definition	Sequencing technique	Sample size	
					Septic	Healthy
**Single datasets**						
Grumaz *et al*. [25]	Grumaz-19	PRJEB21872 PRJEB30958	Sepsis-2	Shotgun	50	–
Grumaz *et al*. [23]	Grumaz-16	PRJEB13247	Sepsis-2	Shotgun	7	15
Gosiewski *et al*. [24]	Gosiewski-17	Requested from authors	Sepsis-1	16S (paired-end)	56	23
Blauwkamp *et al*. [22]	Karius	PRJNA507824	Sepsis-1	Shotgun	117	170
**Combined datasets**						
All single datasets	Pooled	All accessions	Sepsis-1 and Sepsis-2	Shotgun and 16S (paired-end)	230	208
Grumaz *et al*. [23] and Grumaz *et al*. [25]	Grumaz-16/19	PRJEB13247 PRJEB21872 PRJEB30958	Sepsis-2	Shotgun	57	15

**Impact Statement**

In this work, we analysed publicly available metagenomics datasets, comparing the patterns of microbial DNA in the blood plasma of septic patients relative to that of healthy individuals. As a technical contribution to (meta)genomic medicine, we demonstrate the application of a state-of-the-art machine learning technique to computationally identify putative contaminant taxa, which confound metagenomic investigations of blood infections. Additionally, the main contribution of our work is to show that septic infections tend to be polymicrobial rather than unimicrobial in nature. Polymicrobial interactions are known to alter infectious disease progression, severity and the host’s response to treatment. As such, our conclusions justify further work into characterising the microbial component of sepsis, and how it may be leveraged for management of sepsis in a clinical setting.

## Introduction

Sepsis poses a significant challenge to public health and was listed as a global health priority by the World Health Organisation (WHO) in 2017. In the same year, 48.9 million cases of sepsis and 11 million deaths were recorded worldwide [1], having a particular impact in low- and low-to-middle income countries [2].

Current research efforts have predominately focused on understanding the host’s response to sepsis. Indeed, all contemporary definitions of sepsis focus on the host’s response and resulting systemic complications. The 1991 Sepsis-1 definition described sepsis as a systemic inflammatory response syndrome (SIRS) caused by infection, with patients being diagnosed with sepsis if they fulfil at least two SIRS criteria and have a culture-confirmed infection [3]. The 2001 Sepsis-2 definition then expanded the scope of SIRS to include more symptoms [4]. More recently, the 2016 Sepsis-3 definition sought to differentiate between mild and severe cases of dysregulated host responses, describing sepsis as a life-threatening organ dysfunction as a result of infection [5]. Significant progress has been made in understanding how dysregulation occurs [6] and the long-term impacts of sepsis [7, 8]. Additionally, early-warning tools have been developed based on patient healthcare records [9–11] and clinical checklists [12, 13]. However, the focus on the host component of sepsis may overlook the important role of microbial composition in the pathogenesis of the disease.

Due to the severity of sepsis, current practice considers identification of a single pathogen sufficient to warrant a diagnosis, without consideration of other, potentially relevant, species in the bloodstream. Upon diagnosis, infections are rapidly treated with broad-spectrum antibiotics. However, blood cultures, the current recommended method of diagnosis before antimicrobial treatment [14], are known to yield false negatives due to certain microorganisms failing to grow in culture [15], particularly in samples with low microbial loads [16]. Culture-based methods, while useful in a clinical context, may therefore under-estimate the true number of causative pathogens infecting septic patients.

Sepsis is a highly heterogeneous disease which consists of both a host component and a microbial component. While the former has been widely studied, the latter appears to represent a largely untapped source of information that could further advance our understanding of sepsis. Several diseases manifest as a result of interactions in a polymicrobial community. For example, microbial interactions in lung, urinary tract and wound infections are all known to contribute to differing disease outcomes (reviewed by Tay *et al.* [17]). These findings suggest that the microbial component of sepsis may also be crucial to understanding its pathogenesis.

Current technologies to investigate the presence of polymicrobial communities have some major limitations. As noted previously, culture-based methods have a high false negative rate. Furthermore, without knowledge of the range of microorganisms that infect blood, co-culture experiments to study microbial interactions prove difficult. For PCR-based technologies, the use of species-specific primers (e.g. SeptiFast [18]) necessitates a priori knowledge of microbial sequences endogenous to septic blood. Lastly, metagenomic sequencing is ubiquitously prone to environmental contamination. This can include DNA from viable cells introduced during sample collection, sample processing or DNA present in laboratory reagents [19–21]: the so-called ‘kitome’. As such, it can be difficult to determine which microorganisms are truly endogenous to the sample, and at what abundance.

In this study, we sought to expand our understanding of the full microbial component of sepsis. Multiple statistical and state-of-the-art machine learning techniques were applied to metagenomic sequencing data published by Blauwkamp *et al.* [22] (henceforth Karius study) from 117 sepsis patients and 170 healthy individuals. To circumvent the problem of potential contamination in metagenomic data, we developed and applied a novel computational contamination reduction technique. We also externally validated our findings using external hold-out datasets comprising three other independent sepsis cohorts. Taken together, our results provide strong evidence for a polymicrobial signature of sepsis and the utility of metagenomic sequencing for the investigation of blood-borne infections.

## Methods

### Datasets

Our primary analysis involved published shotgun metagenomic sequence data from the Karius study [22]. As detailed in this study, patients were diagnosed with sepsis if they presented with a temperature > 38 °C or < 36 °C, at least one other SIRS criterion and evidence of bacteraemia. Bacteraemia was confirmed via clinical microbiological testing performed within 7 days after collection of the blood samples. The list of pathogens identified by such tests (which we refer to as ‘culture-confirmed’ pathogens) can be found on GitHub (github.com/cednotsed/Polymicrobial-Signature-of-Sepsis/blob/master/datasets/karius_parsed_metadata.csv) and corresponds to Table S5 in the Karius study. This included tissue, fluid and blood cultures, serology, and nucleic acid testing. The clinical outcome of each patient was not reported in the original study. Seven of the 117 septic patients were found to have more than one ‘culture-confirmed’ pathogen identified by microbiological testing (Table S5 in the Karius study). According to the Karius study, healthy individuals were ‘screened for common health conditions including infectious diseases through a questionnaire and standard blood donor screening assays’. We believe this to be reasonable grounds for ruling out bloodstream infections in healthy patients (*i.e.* of non-septic origin).

To determine if the findings of our primary analysis were applicable beyond the Karius dataset, we also used metagenomic sequencing data from three other independent sepsis cohorts [23–25], where participants were recruited under different sepsis definitions, and samples were sequenced using different sequencing strategies (single datasets; Table 1). All four datasets were combined to yield the *Pooled* dataset (combined datasets; Table 1), which was used to determine if models could perform well given data from diverse sources. To further test the generalizability of our models, we held out one dataset and used it to evaluate models trained on the remaining datasets (see section ‘*Holdout cross-validation*’). Since Grumaz-16 did not contain samples from healthy individuals, it had to be combined with Grumaz-19 to form a single holdout dataset named Grumaz-16/19 (combined datasets; Table 1). In this case, Karius and Gosiewski-17 were used for model training and optimization while Grumaz-16/19 was used for evaluation. We will henceforth refer to each dataset by its dataset alias as shown in Table 1.

### Data pre-processing

As described in the Karius study, input circulating free DNA was sequenced using a NextSeq500 (75-cycle PCR, 1×75 nt). Raw Illumina sequencing reads were demultiplexed by bcl2fastq (v2.17.1.14; default parameters) and quality trimmed using *Trimmomatic* (v0.32) [26] retaining reads with a quality (Q-score) above 20. Mapping and alignment were performed using *Bowtie* (v2.2.4) [27]. Human reads were identified by mapping to the human reference genome and removed prior to deposition in NCBI’s Sequence Read Archive (PRJNA507824).

For Grumaz-16 and Grumaz-19, *BBMap* (v38.79) [28] was used to trim adapter sequences, remove reads with a Q-score below 20 and remove reads mapping to a masked human hg19 reference (https://tinyurl.com/yya4xmrg). For the Gosiewski-17 dataset, we performed the same pre-processing steps as reported in the associated study [24]. Briefly, primers and adapters were removed using *Cutadapt* (v1.18) [29], paired reads were merged using *ea-utils* (v1.1.2.537) [30], merged reads and forward unmerged *fastq* files were concatenated, and reads with a Q-score below 20 were removed using *BBMap*.

Taxonomic classification of all shotgun sequencing data was performed using *Kraken 2* (v2.0.9-beta; default parameters) [31]⁠ with the *maxikraken2_1903_140* GB database (https://tinyurl.com/y7zfg9kr). To mitigate potential misclassification of closely related species (*e.g. Escherichia coli* and *Shigella* species) during taxonomic assignment, we considered only microbial abundance at the genus rank for downstream analyses. For the Gosiewski-17 dataset, *Kraken 2* with a *Kraken 2*-built *Silva* database was used instead of conventional 16S amplicon metagenomic classification methods [32]. Read assignments for all ‘culture-confirmed’ bacterial pathogens using the *maxikraken2_1903_140* GB and *Kraken 2*-built *Silva* databases are shown in Fig. S1. While the relative number of reads assigned to each bacterial genus showed some inconsistencies, this hardly affected the classifier performance of septic and healthy patients (Fig. S2). This suggests that our model is fairly robust to heterogeneity which may be introduced by the classification step. For downstream analyses, we use the genera assignments based on the *Kraken 2*-built *Silva* database for the 16S Gosiewski-17 samples. Additionally, all unclassified reads were excluded from the analyses.

Unexpectedly, for the Karius dataset, a small number of reads were assigned to the genus *Homo,* which was possibly due to misclassification. Mapping of all reads in the Karius sequencing data found just 873 bases with 96 % identity to a masked human hg19 reference (https://tinyurl.com/yya4xmrg), with an average of 0.3 reads per sample (range: 0–7 reads). Since human reads were already removed in the bioinformatics workflow of the Karius study, we did not perform an additional human read removal step to avoid introducing biases in the data.

The ouput of taxonomic assignment is a data matrix with samples represented in rows and taxa in columns (*i.e.* features). Each element in the matrices represented the total number of reads assigned to each taxon, which we loosely refer to as ‘abundance’. The set of taxa used in each analysis will henceforth be referred to as the ‘feature space’. Where a single dataset was used to train a single model, the feature space comprised all microbial taxa identified during taxonomic assignment. Where multiple datasets were used in tandem to train a single model, the feature space comprised the microbial taxa common to all datasets. Feature spaces that have not undergone any statistical removal of microbial taxa are denoted by *Neat*.

### Model training, optimization and nested cross-validation

To assess the suitability of taxonomic assignments for discriminating between septic and healthy blood metagenomic samples, gradient-boosted tree classifiers were trained and evaluated using the data matrices parsed from the *Kraken 2* taxonomic assignments. The task of all classifiers was to predict if a sample belonged to a septic or healthy individual given the read counts assigned to microbial taxa. Classifiers were trained with a binary-logistic loss function and implemented using *XGBoost* API (v0.90) [33]. Model optimization was performed using a randomised hyperparameter optimization protocol [34] with 1000 samples, implemented using *RandomizedSearchCV* in the *Scikit-learn* API (v0.23.1) [35]. The test error of each model was estimated using a nested, stratified, 10×10-fold cross-validation procedure. Nested cross-validation was necessary to obtain an unbiased estimate for test error since hyperparameter optimisation was required [36]. Briefly, in each iteration of the outer cross-validation loop, a tenth of the data is held-out. The remaining data are used in an inner cross-validation loop where a search for the best set of hyperparameters is performed. The held-out data in the outer loop are then used to evaluate the model with the best set of hyperparameters identified in the inner loop. Separately, a hyperparameter optimisation protocol was performed using the entire dataset, yielding the hyperparameter set that maximises the receiver operating characteristic curve (AUROC) metric. This hyperparameter set was then used for downstream analyses.

### Holdout cross-validation

To determine if our models were generalisable across the different sepsis cohorts, the data from three of four sepsis cohorts were combined for model training and hyperparameter optimisation. The test error of each optimized model was then estimated using the holdout dataset. We refer to this protocol as ‘holdout cross-validation’. For holdout cross-validation, precision, recall and the area under the precision-recall curve (AUPRC) were used as performance metrics since they are more informative when used on imbalanced test sets [37]. Any statistical filtering of features (see sections ‘*SHAP decontamination*’ and ‘*Simple decontamination*’) was performed before model evaluation.

### Model interpretation

To interpret models, each feature in a single sample was assigned a SHAP (SHapley Additive exPlanations) value, which corresponds to the change in a sample’s predicted probability score (*i.e.* probability of sepsis) when the feature is either present or absent. Using SHAP values therefore allows the decomposition of predicted probability scores for each sample into the sum of contributions from individual genera. The relative importance of each feature was inferred via its mean absolute SHAP value across all samples. A higher mean absolute SHAP value implies that the feature has a larger impact on the model predictions. SHAP values were computed using *TreeExplainer*, part of the *shap* library (v0.34.0) [38]. For every model, SHAP values were computed for the whole dataset by setting the *feature_pertubation* parameter to ‘interventional’.

### SHAP Decontamination

SHAP Decontamination was performed in two main steps. First, genera that are not currently identified as known human pathogens were removed. This selection was based on a study by Shaw *et al.* [39], who considered a ‘human pathogen’⁠ to be any microbial species for which there is evidence in the literature that it can cause infection in humans, sometimes in a single patient. The list of known human pathogens used can be found on GitHub (https://github.com/cednotsed/Polymicrobial-Signature-of-Sepsis/blob/master/datasets/pathogen_list.csv) and was downloaded from FigShare [40]. Second, a classifier was optimised and trained on genera abundance (*Neat* feature spaces). SHAP values for model predictions on the dataset were then calculated. Genera with a negative Spearman’s correlation between their corresponding SHAP values and abundances were removed. Spearman’s correlations were calculated using *spearmanr* as part of the *SciPy* library (v1.4.1) [41]. A new classifier was then retrained using the previously optimized set of parameters but with this new reduced feature space. This process was repeated iteratively until the number of genera retained remained constant. The resultant feature space is denoted by *CR*.

To test the hypothesis that genera containing true pathogens are positively associated with sepsis, we inspected the SHAP values and read counts assigned to the genera corresponding to cases of each type of ‘culture-confirmed’ infection (e.g*.* SHAP value/read count assigned to *Escherichia* for only *Escherichia *-positive samples) using the *Karius-Neat* feature space. The SHAP values were all greater or equal to zero apart from a single sample which had a negative SHAP value for *Mycobacterium* (Fig. S3). The assigned read counts were non-zero except for one sample with a ‘culture-confirmed’ fungal *Candida glabrata* infection reported (SRR8288759). These findings suggest that SHAP values can successfully recover experimentally identified pathogens.

### Simple Decontamination

We also employed a more direct, model-free contaminant removal technique (Simple Decontamination) that follows the same underlying premise of SHAP Decontamination. In this procedure, genera in the *Neat* feature space that were significantly (*P* < 0.05) more abundant in healthy controls than septic samples were considered contaminants and removed. The resultant feature space is denoted by *SD*.

### Microbial networks

Microbial co-occurrence networks were constructed using the *SparCC* algorithm [42], implemented in the *SpiecEasi* package (v1.1.0) [43] and visualized using *Igraph* (v1.2.5) [44]. *SparCC* was used to account for compositionality that could lead to spurious correlations. Separate networks were constructed for the genera assignments of septic and healthy metagenomes. To determine the microbial associations present exclusive to septic samples, a corrected sepsis network was produced. This network was constructed by subtracting all edges of the healthy network from the sepsis network. Only co-occurrence relationships where the *SparCC* correlations exceed 0.2 were retained. The *Karius-SD* feature space was used as input.

## Results

### Metagenomic sequencing can be used to discriminate septic from healthy samples

The performance of all classifiers is summarized in Table 2. Models were first trained and evaluated using 117 septic patients and 170 healthy individuals in the Karius study (Table 1). Classifiers could discriminate between sepsis from healthy samples using the read counts assigned to each microbial taxon (*Karius-Neat* model; AUROC=0.995). Classifiers performed similarly well when using a more diverse dataset comprising data pooled from all four sepsis cohorts (*Pooled-Neat* model; AUROC=0.982). We also tested the effect of normalising assigned read counts by the total per-sample count. Such normalisation resulted in reduced classification performance (*Karius-normalised* model; AUROC=0.943) and so was not performed for the rest of the models tested.

**Table 2.** Summary of models trained Models were optimized and evaluated via a nested cross-validation protocol. The prefix and suffix of each model name corresponds to the dataset and contamination reduction technique applied, respectively. *Neat*, *SD* and *CR* refer to the feature spaces with no decontamination, Simple Decontamination, and SHAP Decontamination applied, respectively (see Methods). *Karius-Without* corresponds to the SHAP-decontaminated feature space after claimed ‘culture-confirmed’ pathogens are excluded. *Karius-Only* refers to the feature space containing only genera with ‘culture-confirmed’ pathogens as features.

No. of features	Feature space	Model performance		
		Precision	Recall	AUROC
1564	*Karius-Neat*	0.976	0.983	0.995
1564	*Karius-normalised*	0.956	0.932	0.943
111	*Karius-SD*	0.896	0.787	0.942
25	*Karius-CR*	0.883	0.810	0.942
22	*Karius-Without*	0.803	0.727	0.915
22	*Karius-Only*	0.929	0.862	0.950
685	*Pooled-Neat*	0.950	0.939	0.982
21	*Pooled-CR*	0.870	0.796	0.904

### SHAP can be used to remove putative sequencing contaminants

Accurate characterization of the microbial component of sepsis requires discrimination between a true biological signal and that arising from putative environmental contamination in metagenomes. We developed and applied a procedure to remove biologically irrelevant genera from the feature space, which we refer to as SHAP Decontamination (CR; see Methods). Briefly, we leveraged SHAP – a state-of-the-art machine learning technique for interpreting ‘black-box’ classifiers [38] – to determine how the read counts assigned to a genus (*i.e.* feature) influence model predictions for each sample. In doing so, we selectively removed putative contaminants from the feature spaces obtained from taxonomic classification. We illustrate this for a single ‘culture-confirmed’ *E. coli *-positive sample in the Karius dataset (Fig. S4).

To evaluate the effectiveness of this approach, we compared SHAP Decontamination to a simpler statistical method for the removal of putative pathogens, which we call Simple Decontamination (SD; see Methods). For the Karius dataset, application of SHAP Decontamination resulted in a pruned feature space of 25 genera while Simple Decontamination resulted in 111 genera. The resultant *Karius-CR* and *Karius-SD* feature spaces, respectively, shared 21 genera in common. Classifiers trained on either of the *Karius-CR* or *Karius-SD* feature space had similarly high performance (Table 2, *Karius-CR/SD*; AUROC=0.942), despite the large reduction in the number of features. This suggests that computational decontamination efficiently removes redundancy in the metagenomic feature space. Furthermore, SHAP Decontamination appears to be more efficient, as demonstrated by the equivalent classification performance, but higher number of removed putative contaminant genera than Simple Decontamination.

**Fig. 1.**
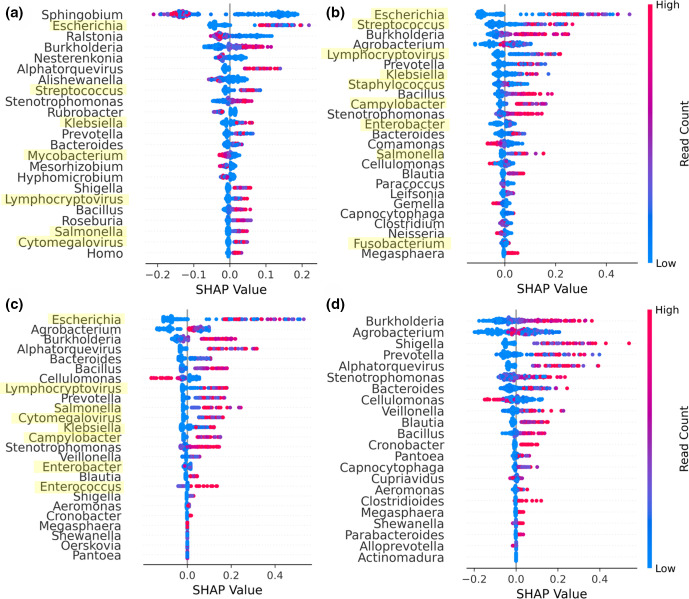
Model interpretation and performance. (**a**) Plot summarizing the SHAP values across all samples for the most important features ranked by the mean absolute SHAP value (highest at the top) for *Karius-Neat*, (**b**) *Karius-SD*, (**c**) *Karius-CR* and (**d**) *Karius-Without* models. Each point represents a single sample. Points with similar SHAP values were stacked vertically for visualization of point density and were coloured according to the magnitude of the feature values (i.e*.* read counts). Genera that contained ‘culture-confirmed’ pathogens are highlighted in yellow.

Separately, we observed that the *Karius-CR* model comprised almost all genera associated with sepsis at higher abundance. Additionally, genera such as *Sphingobium *, *Mesorhizobium* and *Ralstonia* were highly important features in the *Karius-Neat* feature space (Fig. 1a), though not present in either the *Karius-SD* or the *Karius-CR* feature space (Fig. 1b, c). These genera are likely to be contaminants since they contribute negatively to the predicted probability of sepsis at high abundance, and have been previously ascribed as common sequencing contaminants [19]. Of the 25 genera in the *Karius-CR* feature space, eight corresponded to genera containing clinically ‘culture-confirmed’ pathogens (see Methods). Notably, *Escherichia* and *Enterobacter *, which are both ‘culture-confirmed’ pathogens but also common contaminants [19], were retained in both decontaminated feature spaces. These findings collectively suggest that computational decontamination procedures were removing putative contaminants while selectively retaining biologically important genera.

### Evidence for a polymicrobial community

**Fig. 2.**
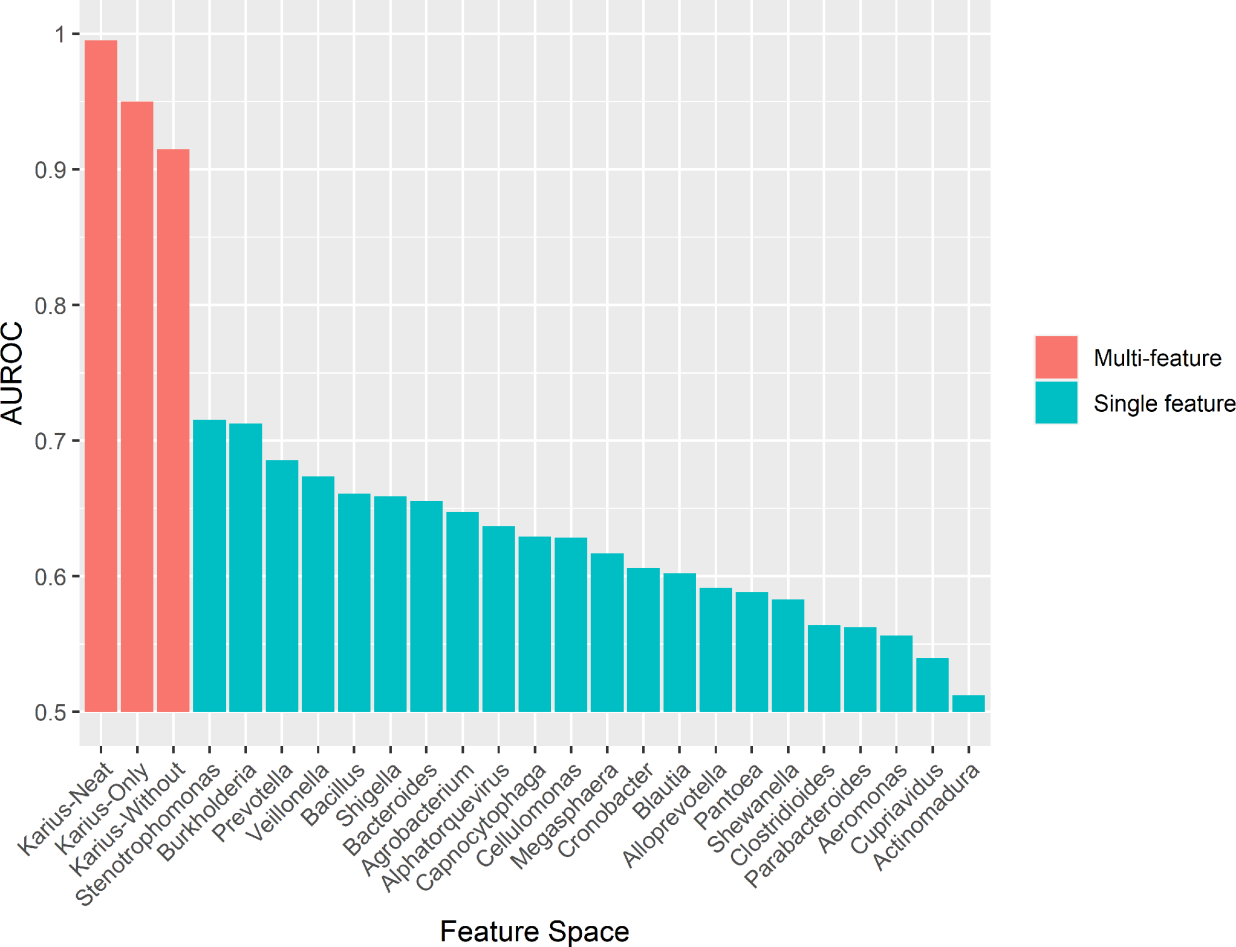
Comparison of performance (AUROC) for the multi-feature models (*Karius-Neat, Karius-Only, Karius-Without* feature space) and single-feature models (*x*-axis). Models were optimised and evaluated using the nested cross-validation protocol.

Having assessed the biological relevance of microbial predictors of sepsis, we provide several pieces of evidence supporting a polymicrobial model of sepsis; that is, that there are sets of microbial genera that delineate septic from healthy blood metagenomes, rather than just individual pathogens. Most notably, a classifier trained on the Karius dataset using the SHAP-decontaminated feature space but with all genera containing clinically identified pathogens (henceforth ‘culture-confirmed’ pathogens; see Methods) removed performed well (*Karius-Without* model; AUROC=0.915), suggesting the presence of these species alone does not capture the full microbial signal of sepsis. Visualization of the SHAP values for this model (Fig. 1d) confirmed that most genera had positive associations with sepsis at higher abundances. To test if any single features in the *Karius-Without* model were driving the high classification performance, we trained and evaluated multiple single-feature classifiers with each genus in the *Karius-Without* feature space. Additionally, we trained a classifier on genera containing ‘culture-confirmed’ pathogens as features only (*Karius-Only*). Fig. 2 shows the performance of the multi-feature *Karius-Neat*, *Karius-Without* and *Karius-Only* models compared to single-feature models. All multi-feature models performed better than those relying on single-feature models.

We then trained classifiers on the pooled dataset to determine if our results were unique to the Karius dataset or whether they were portable to other sepsis cohorts. Current metagenomics datasets are limited in their suitability for external validation due to the use of different sequencing technologies, differing sepsis definitions and small sample sizes. However, despite the pooled dataset comprising multiple data sources from different studies, the classifier still performed well (*Pooled-Neat* model, AUROC=0.982; *Pooled-CR* model, AUROC=0.904). This suggests strongly that there is a generalisable microbial signature which can be leveraged across metagenomic datasets.

**Fig. 3.**
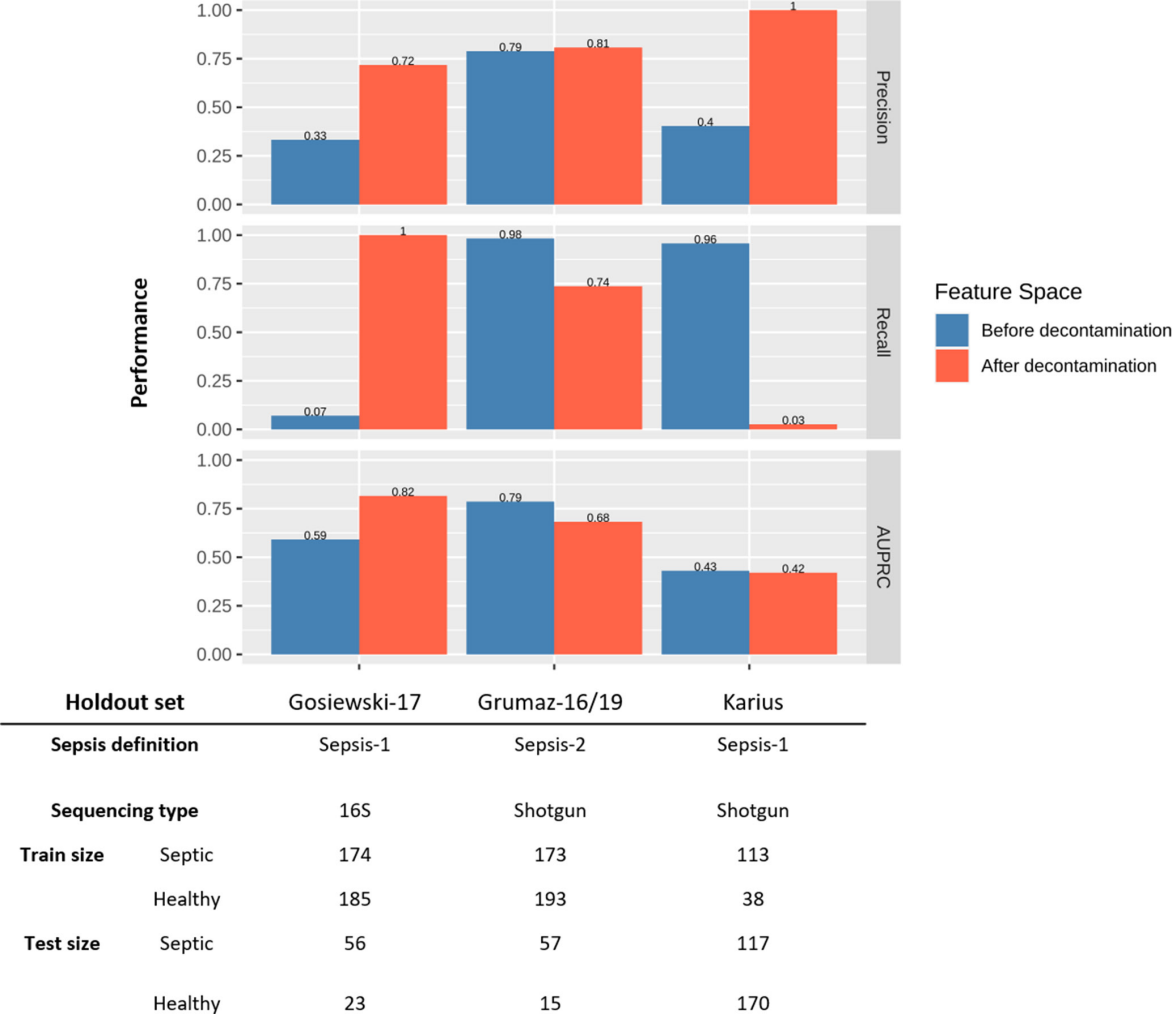
Generalisability of models across sepsis cohorts. Model performance before and after SHAP Decontamination determined via holdout cross-validation (see Methods). The table appended describes the sepsis definition used, sequencing type and test size for each holdout dataset, and the corresponding size of the training data.

To more formally test the generalisability of the observed polymicrobial signature, we used holdout cross-validation (see Methods). Most notably, the classifier trained on shotgun metagenomic data and tested on 16S data as the holdout set (Gosiewski-17) did not perform well. However, after SHAP Decontamination, classification performance improved markedly. Interestingly, this performance increase was not observed when using the other datasets as holdout sets (Fig. 3). Indeed, the classifier trained with Grumaz-16/19 as the holdout set performed well before SHAP Decontamination, but relatively worse after. Additionally, holding out the Karius dataset resulted in poor classification performance both before and after SHAP Decontamination. A possible explanation for SHAP Decontamination lowering classification performance when Grumaz-16/19 is used as the test set is that septic cases recruited in these studies were based on different sepsis definitions, which may involve a different set of pathogens and reflect different aetiologies. Separately, the poor performance observed when the Karius dataset is used as the test set can be attributed to the highly imbalanced training dataset (Fig. 3).

**Fig. 4.**
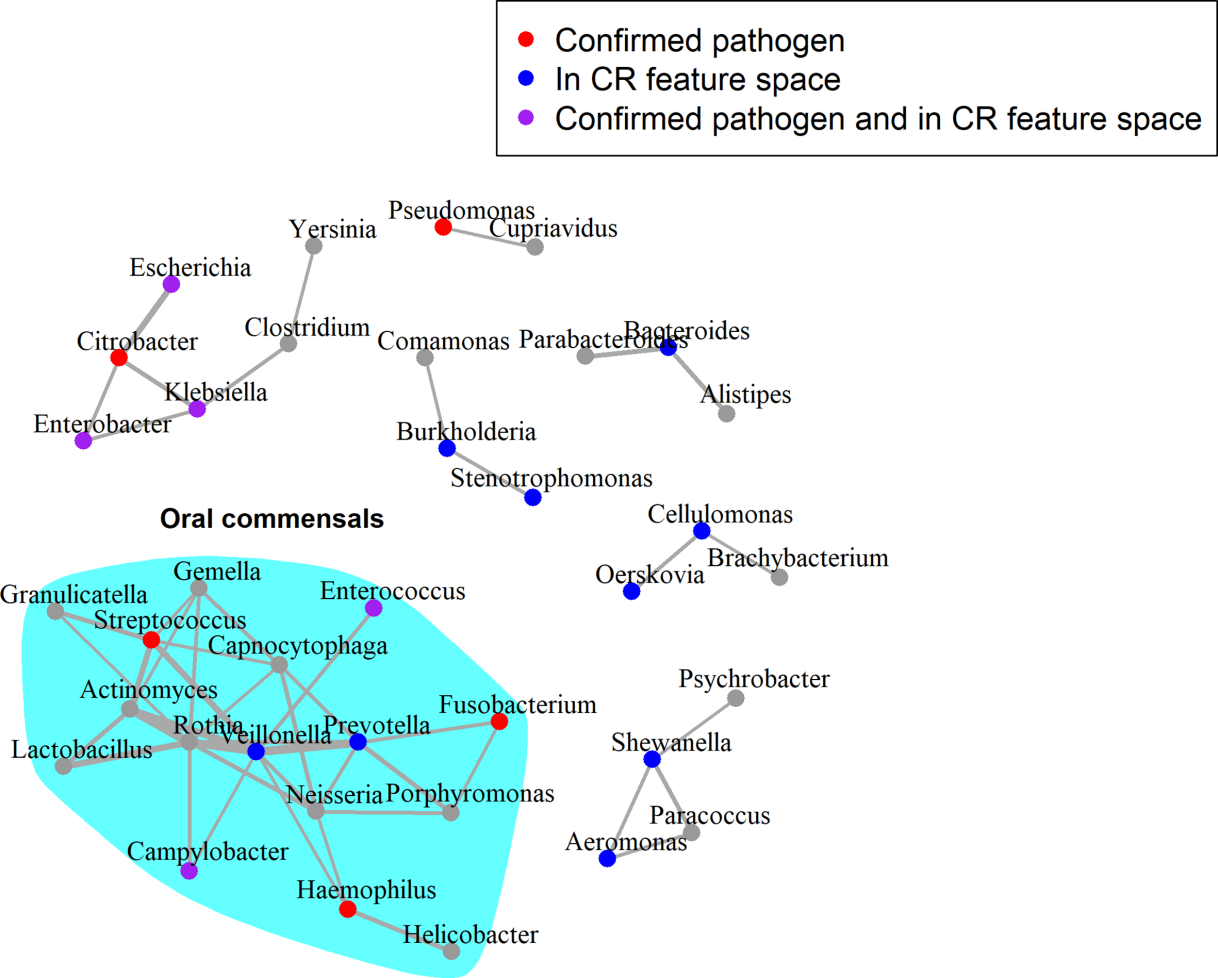
Corrected microbial co-occurrence network for genera assigned in sepsis metagenomes. Input data correspond to the *Karius-SD* feature space. The edges in this network represent those in the septic network that were not present in the healthy network. The widths of edges are weighted by the strength of the *SparCC* correlations. Nodes are coloured as per the legend at the top, with ‘culture-confirmed’ pathogens those experimentally shown to be implicated in sepsis. The layout of the graph was generated using the Fruchterman–Reingold algorithm.

Lastly, microbial co-occurrence networks were used to identify relationships between genera that were exclusive to samples from septic patients. Two genera are said to co-occur if an increase in the abundance of one is associated with an increase in the abundance of the other. The presence of such relationships would lend weight to the polymicrobial nature of sepsis infections. The *Karius-SD* feature space was used in this analysis to corroborate previous analyses using the *Karius-CR* feature space. Multiple co-occurrence relationships between genera were present in the corrected network including those containing 10 of the 22 ‘culture-confirmed’ pathogens and 14 of the 25 genera in the *Karius-CR* feature space (Fig. 4). Interestingly, we detected a group of co-occurring genera associated with the oral cavity (Fig. 4), as suggested by the Human Oral Microbiome Database [45] (accessed 15 July 2020) and the current literature [46–49]. This was also present in the corrected network when the *Pooled-SD* feature space was used as input (Fig. S5).

## Discussion

### The polymicrobial signature of sepsis

Our work demonstrates a clear polymicrobial signal in sepsis, where multiple, co-occuring, genera can be used to discriminate blood metagenomes of septic patients from that of healthy controls. The high performance of the *Karius-Only* model highlights that genera containing ‘culture-confirmed’ pathogens were very useful in delineating septic from healthy samples. More importantly, the *Karius-Without* model, which had these genera removed, also performed well, suggesting that the abundance of microbial genera that were not amongst the ‘culture-confirmed’ pathogens are also highly relevant to delineate septic from healthy samples. Furthermore, the single-feature models performed poorly, highlighting that no genus is solely responsible for the high classification performance of the *Karius-Without* model, further supporting the polymicrobial nature of sepsis infections.

We also demonstrate that the polymicrobial signal we detected is generalisable across datasets, first by nested cross-validation with all datasets pooled (*Pooled-CR* model) and then with holdout cross-validation using the Gosiewski-17 or Grumaz-16/19 datasets as test sets. The increased performance after SHAP Decontamination when holding out 16S data (Gosiewski-17) suggests that the retained set of genera allow a markedly more generalizable decision boundary to be learnt, even across sequencing techniques.

Additionally, the multiple co-occurrence relationships between genera detected suggest that there may be a distinct microbial community that tends to be present during sepsis infection. Although our networks were inferred computationally, published evidence supports possible synergies between some of the co-occurring genera we detected. For example, *Stenotrophomonas* and *Burkholderia* are known to play a collective role in the pathogenesis of cystic fibrosis [50]. Additionally, *Klebsiella pneumoniae* was found to be able to transmit extended-spectrum beta-lactamase genes to *Citrobacter freundii* and *E. coli* [51], potentiating synergism during polymicrobial infections. Furthermore, using fluorescence *in-situ* hybridisation, interspecies spatial associations were found between *Prevotella *, *Veillonella *, *Streptococcus *, *Gemella *, *Rothia* and *Actinomyces* in dental biofilms [52]. The tendency of bacteria of these genera to aggregate in biofilms agrees with the their strong correlations in the corrected sepsis network (Fig. 3). Moreover, bacterial cells from the genera *Prevotella* and *Actinomyces* were found to be in contact with the most number of bacterial species, suggesting that they were key players in maintaining intercellular adhesion and hence biofilm maturation in the oral cavities [52]. This was recapitulated in the corrected sepsis network, where the two genera are central nodes in the oral commensal cluster (Fig. 3). These examples suggest that the co-occurrence relationships we computationally detected may reflect genuine biological relationships.

Notably, the presence of a densely connected cluster of oral colonisers — some of which were identified to have interspecies spatial associations [52] – may point to a potential reservoir of sepsis pathogens. This also suggests the possibility of opportunistic infections from the human microbiota and dysbioses that could affect disease severity, given that oral infections are a known risk factor for systemic disease [53, 54]. This hypothesis is in line with the reported changes in nasal microbiomes in septic individuals [55] and the associations of intestinal dysbiosis with increased susceptibility to sepsis [56]. If these hypotheses were validated, the microbiome profiles of patients might offer opportunities to assess a patient’s risk of developing sepsis prior to onset. Further investigation of the interactions between different clusters of genera in the corrected sepsis network, together with expanding our analyses to future datasets, may yield valuable insights into the underlying biology of sepsis infections and ultimately inform treatment.

### The need to account for environmental contamination

Contamination from environmental sources poses one of the greatest challenges for metagenomic investigations of microbial communities, particularly in low-biomass and clinical samples [20, 57]. It is therefore crucial to discriminate between contaminants and biologically relevant taxa and to remove putative contaminants to protect against spurious signals.

The main premise behind SHAP Decontamination is that pathogens should occur at higher abundance in septic patients relative to healthy controls. This is because we expect most infections to be characterised by the proliferation of microorganisms [58, 59] and, as such, true pathogenic genera should contribute to a higher predicted probability of sepsis at higher abundances. Consequently, the abundance of contaminant taxa would demonstrate a negative Spearman’s correlation with their corresponding SHAP values. This allows putative contaminant genera to be computationally detected and removed. Our results demonstrate the efficacy of our post-hoc contamination reduction technique called SHAP Decontamination in removing redundancy in the feature space while selectively retaining taxa involved in sepsis. It is likely that the taxa removed in this procedure would in principle include commensals and environmental contaminants introduced during sample collection or preparation. As such, application of this technique provides greater confidence that the polymicrobial signals we observed were not largely driven by contaminants.

We appreciate that a more rigorous evaluation of this technique, particularly with mock communities, will be required. As an alternative to our contamination reduction technique, statistical decontamination techniques identifying inverse relationships between the assigned abundance of taxa and sample DNA concentration [60, 61] could be used. However, this method was not applicable for our study since the sample DNA concentrations in the datasets used were not reported.

### Potential for metagenomics-based diagnostics

Although we do not claim to have developed a model sufficiently robust for immediate diagnostic purposes, our results highlight the clear promise of metagenomics-informed diagnostic models, which have also been suggested by previous studies [22, 62, 63]. To put the high performance of our models in context, Mao *et al.* [9] reported that InSight, a model trained on vital signs of patients, had a diagnostic AUROC of 0.92 using Sepsis-2 as the ground truth. They also reported that the Modified Early Warning Score (MEWS), Sequential Organ Failure Assessment (SOFA) and SIRS had an AUROC of 0.76, 0.63 and 0.75 respectively. Additionally, a classifier trained on nasal metagenomes of septic and healthy samples had an AUROC of 0.89 with Sepsis-3 as the ground truth [55]. Notably, it is difficult to compare the performance of models trained with labels generated by different definitions of sepsis, which is also inherently a highly heterogeneous disease. Further, the discrepancies in model performance could be due to differences in the size of training and testing datasets. At the very least, our results suggest that the microbial component of sepsis alone contains sufficient information for the diagnosis of sepsis. A crucial next step will be to generate larger datasets, from more diverse sources, to allow the training of more robust and generalisable models for diagnostic or prognostic use.

### Limitations

We identified several limitations in our study. First, metagenomic sequencing involves measurements of circulating free DNA and not of viable microorganisms in blood. As such, the detection of DNA from multiple taxa does not necessarily represent the true number or abundance of active taxa present. However, multiple studies have demonstrated high concordance of targeted [64] or shotgun metagenomic sequencing with culture [22, 62, 65]. This suggests some level of agreement between the presence of microbial cells and their DNA in blood. Additionally, given its higher sensitivity and throughput, metagenomic sequencing appears to be the best tool currently available for gaining insights into polymicrobial infections.

Though our results suggest the importance of multiple genera in delineating metagenomes of septic patients from that of healthy controls, the aetiological contributions of these genera and their ecological relationships cannot be inferred. Such hypotheses must be confirmed experimentally. It is also important to keep in mind that the models presented in this study are not prognostic in nature, in that they were not trained to predict the onset or progression of sepsis. However, furthering our understanding of the microbial component of sepsis may prove useful in the development of better prognostic tools.

Some genera such as *Escherichia* and *Enterobacter* contain both biologically relevant genera and common sequencing contaminants. As such it is expected that a proportion of DNA molecules, and hence sequencing reads, may have come from contamination rather than microorganisms endogenous to blood. The abundance of these microorganisms, as detected by metagenomic approaches, may differ from the true abundance.

Additionally, *k*-mer-based approaches may be less accurate for taxonomic classification compared to, for example, Bayesian sequence read-assignment methods [66]. As such, we used taxonomic assignments at the genus level which were shown to be, in general, more reliable than that at the species level [67]. We also appreciate that *k*-mer-based classification approaches are significantly faster [68], which may provide clinically relevant turnaround times that are important in sepsis diagnostics.

Finally, we acknowledge the relatively small size of the datasets used in our analyses. As a result, the models presented in this study are not yet robust enough to be used in a clinical context. A larger and more diverse dataset is required to develop such models. This is to ensure that models can learn a more generalisable decision boundary for accurate sepsis diagnosis.

Irrespective of these limitations, our results nonetheless demonstrate the importance of considering the full polymicrobial component of sepsis and suggest that a metagenomics-based approach may provide biological and clinical insights supporting the future development of rapid diagnostic tools.

### Future directions and implications of polymicrobial sepsis

A major next step forward would be to elucidate the functional role of the polymicrobial communities we identify in sepsis. One key hypothesis is whether there are different clusters of microbial communities in different sepsis aetiologies. Evidence for discriminatory microbial signals in different manifestations of disease would facilitate sepsis to be redefined to also include a microbial component. However, to robustly test such hypotheses, a much larger sepsis cohort must be recruited to provide adequate statistical power, particularly considering the true number of sepsis subtypes is unknown. The associations between detected polymicrobial communities and disease severity could also be investigated. To do so, anonymised healthcare records with detailed curation of the clinical outcomes and treatment history of each patient would be required. In addition, pre-infection data from animal models holds promise to identify taxa relevant for the early detection of sepsis, which can be an important bottleneck to good patient outcomes.

It would also be valuable to investigate how identified polymicrobial communities may change during the course of infection. This can be done via analysis of microbial community dynamics [69] using longitudinal metagenomic sampling. By monitoring the change in microbial composition along the course of infection, ecological relationships between pathogens can be inferred. Additionally, this would allow for a better identification of key taxa involved in sepsis at the level of the microbial species together with the presence of particular antimicrobial resistance genes. Lastly, co-culture experiments [70] could be performed to elucidate interactions between pathogens. These could also be paired with metabolomic approaches, which may be useful in identifying possible synergies or antagonisms between microbial species [69, 71, 72].

The advent of large-scale metagenomic sequencing of clinical samples offers new opportunities to better characterize the pathogens contributing to systemic infections, and unlike culture-based methods are not limited to organisms that are fast-growing or culturable. In this study, we demonstrate the promise of a metagenomics-based approach to sepsis. Our results provide evidence that septic infections should be considered as polymicrobial in nature, comprising multiple co-occurring pathogens indicative of disease. Our findings thus pave the way for more microbial-focused models of sepsis, with long run potential to inform early detection, clinical interventions and improve patient outcomes.

## Supplementary Data

Supplementary material 1
